# Müllerian duct aplasia, renal aplasia, and cervicothoracic somite dysplasia syndrome triad with rare ovarian ectopia on magnetic resonance imaging

**DOI:** 10.11604/pamj.2026.54.30.48987

**Published:** 2026-06-04

**Authors:** Gahana Kataria, Vaishali Dhawan

**Affiliations:** 1 Department of Radio-Diagnosis, Jawaharlal Nehru Medical College, Datta Meghe Institute of Higher Education and Research (DU), Sawangi (Meghe), Wardha, Maharashtra, India

**Keywords:** MURCS syndrome, primary amenorrhea, Müllerian agenesis, ectopic ovary, renal agenesis, magnetic resonance imaging

## Image in medicine

A 30-year-old female presented with primary amenorrhea despite normal secondary sexual characteristics. Physical examination revealed Tanner Stage V breast development and a blind-ending vaginal pouch. Laboratory investigations showed a 46, XX karyotype with functional ovaries. She reported monthly cyclic lower abdominal pain since adolescence. Multiplanar magnetic resonance imaging (MRI) of the pelvis with abdomen revealed a complete müllerian duct aplasia, renal aplasia, and cervicothoracic somite dysplasia (MURCS) syndrome triad. Sagittal and axial T2-weighted images demonstrated a small hypoplastic uterine structure deviated to the left, consistent with severe uterine hypoplasia/unicornuate configuration. Axial T2-weighted imaging showed complete right renal agenesis with compensatory left kidney hypertrophy showing mild hydroureteronephrosis. Coronal T2-weighted sequences revealed an ectopic right ovary in the right iliac fossa, retroperitoneally positioned posterior to the cecum (1.9x2.3x4.4cm with multiple small cysts), while the left ovary maintained normal position (2.2x1.5x2.6cm with multiple follicles). Sagittal spine imaging demonstrated dorsolumbar scoliosis secondary to hemivertebra formation. MURCS syndrome represents the most severe Mayer-Rokitansky-Küster-Hauser (MRKH) syndrome type II phenotype, affecting 1 in 4,500-5,000 females. A retroperitoneal ectopic ovarian position increases the risk of torsion. Recent genetic discoveries identified GREB1L gene mutations in 2.7% of cases. Management requires multidisciplinary care addressing psychological impact, vaginal reconstruction, and fertility options including uterine transplantation. The ectopic ovary requires monitoring for torsion, while the solitary kidney with hydroureteronephrosis mandates lifelong nephroprotective strategies. This case exemplifies the importance of comprehensive MRI evaluation in primary amenorrhea diagnosis.

**Figure 1:**
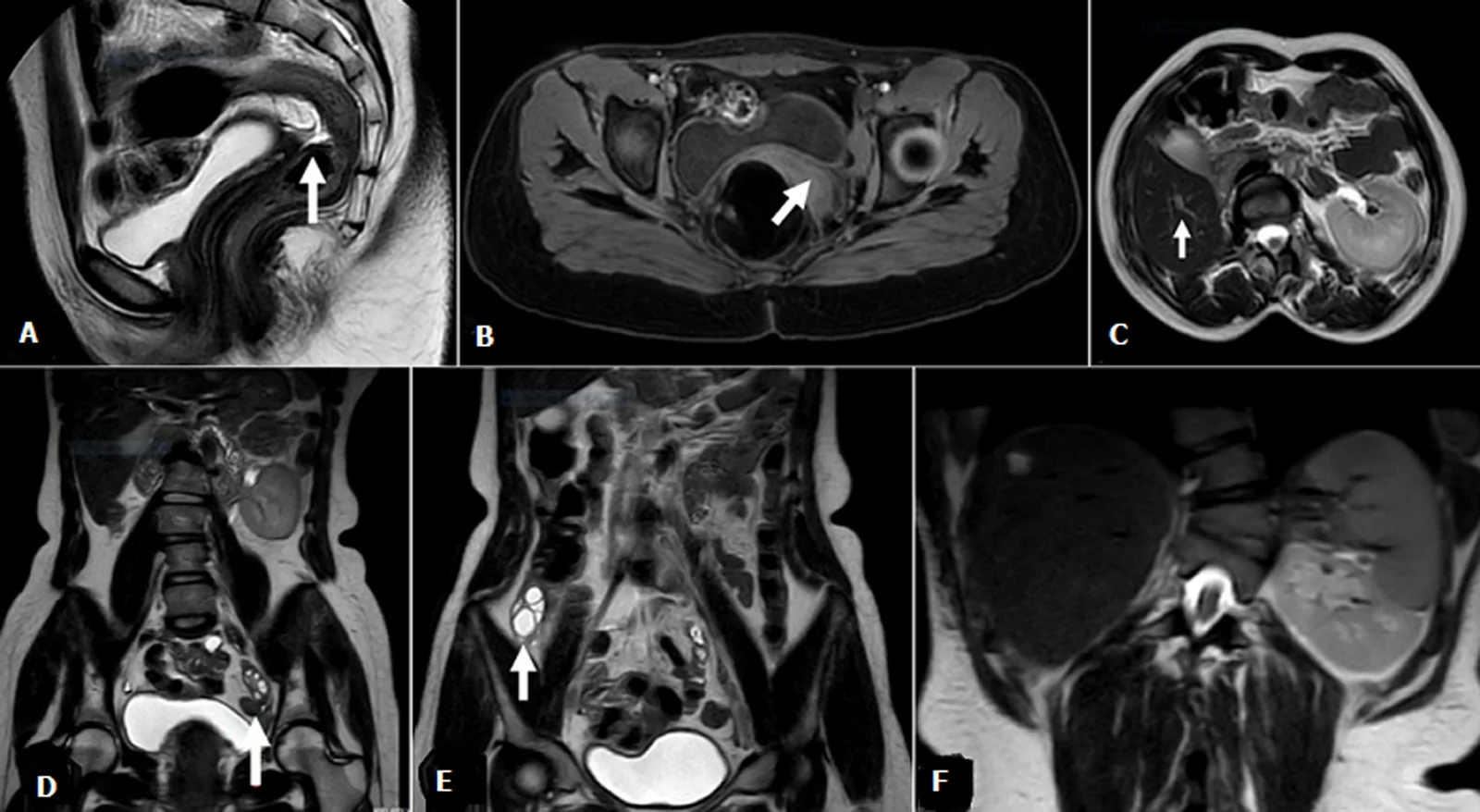
MRI features of MURCS syndrome: A, B) sagittal and axial T2-weighted images show hypoplastic, left-deviated uterus (arrows); C) axial T2 image demonstrates right renal agenesis with compensatory left kidney hypertrophy and mild hydroureteronephrosis (arrows); D, E) coronal T2 images reveal ectopic right ovary in right iliac fossa, retroperitoneal behind cecum (arrow), with multiple cysts, left ovary in normal fossa with follicles; F) sagittal spine image shows dorsolumbar scoliosis due to hemivertebra

